# Investigation of Atrial Vortices Using a Novel Right Heart Model and Possible Implications for Atrial Thrombus Formation

**DOI:** 10.1038/s41598-017-17117-3

**Published:** 2017-12-01

**Authors:** Utku Gülan, Ardan Saguner, Deniz Akdis, Alexander Gotschy, Robert Manka, Corinna Brunckhorst, Markus Holzner, Firat Duru

**Affiliations:** 1 ETH Zurich, Institute of Environmental Engineering, Zurich, 8093 Switzerland; 2 University Heart Center, Department of Cardiology, Zurich, 8091 Switzerland; 3 Center for Integrative Human Physiology, University of Zurich, Zurich, 8091 Switzerland; 4 Institute for Biomedical Engineering, University and ETH Zurich, Zurich, 8092 Switzerland

## Abstract

The main aim of this paper is to characterize vortical flow structures in the healthy human right atrium, their impact on wall shear stresses and possible implications for atrial thrombus formation. 3D Particle Tracking Velocimetry is applied to a novel anatomically accurate compliant silicone right heart model to study the phase averaged and fluctuating flow velocity within the right atrium, inferior vena cava and superior vena cava under physiological conditions. We identify the development of two vortex rings in the bulk of the right atrium during the atrial filling phase leading to a rinsing effect at the atrial wall which break down during ventricular filling. We show that the vortex ring formation affects the hemodynamics of the atrial flow by a strong correlation (*ρ* = 0.7) between the vortical structures and local wall shear stresses. Low wall shear stress regions are associated with absence of the coherent vortical structures which might be potential risk regions for atrial thrombus formation. We discuss possible implications for atrial thrombus formation in different regions of the right atrium.

## Introduction

Blood flow in the right heart is complex and the assessment of hemodynamics within the right-sided cardiac chambers is challenging. Despite the physiologic and pathophysiologic importance of the right heart and disturbed blood flow within the right-sided cardiac chambers in many cardiovascular diseases such as arrhythmogenic right ventricular cardiomyopathy (ARVC), right ventricular ischemia, tricuspid valvular (TV) and pulmonary valvular (PV) heart diseases^1^, studies on the hemodynamics of the right heart have lagged behind those on the left heart^2,3^.

Vortex ring formation is a prominent characteristic of the cardiovascular system and may indicate overall cardiac health^4,5^. It has been shown that diastolic vortex ring features are consistently present in healthy human hearts, whereas a considerable disruption of vortex ring formation was reported to be associated with heart failure^5^. In healthy adults, the right atrium redirects the blood flow and produces coherent rotational patterns during ventricular systole and diastole allowing blood to slingshot into the right ventricle (RV)^6,7^. On the other hand, cardiac hemodynamics in newborn infants are different from those in adults involving a disruption of rotational flow patterns. Although this adaptation is physiological in newborn infants, disruption of rotational flow patterns may be an indicator of circulatory dysfunction in the adult heart^7^. In the athlete’s heart, helical flow patterns and recirculating blood flow are similar to the normal healthy adult’s heart, which implies that the heart is adapting to accommodate vortical flow structures even under strenuous dynamic exercise conditions^8^. Hence, the analysis of vortex formation appears to be a useful tool for assessing pathophysiological changes in the human heart. Right atrial thrombosis formation is rare as compared to thrombus formation in the left atrium, and mechanisms of thrombus formation within the right atrium are not well understood^9,10^. It has been shown that turbulence increases the probability of thrombus formation in the cardiovascular system^11^. Since the presence of turbulence is an indicator in assessing the abnormalities in the circulatory system^12,13^, studies on assessing blood flow need to consider the influence of vortex formation, disturbed flow and turbulence. To the best of our knowledge, such a study has not yet been conducted in the right atrium.Therefore, the aim of this study was to both qualitatively and quantitatively characterize vortex formation, and to assess the temporal and spatial evolution of vortical structures and their association with wall shear stresses within the healthy human right atrium.

## Results

### Qualitative flow analysis

**Figure 1:**
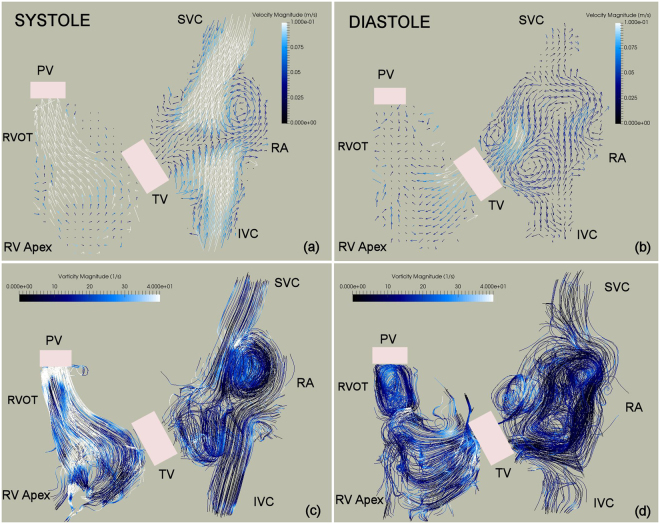
Planar slice of the phase averaged velocity vector map color coded with velocity magnitudes during right ventricular systole (**a**), diastole (**b**) and side view of three dimensional streamlines color coded with vorticity magnitude during right ventricular systole (**c**), diastole (**d**). The white color represents higher velocity magnitudes, whereas black color represents lower velocity magnitudes which are typically associated with lower velocity gradients.

The phase averaged velocity vector map, color coded with velocity magnitudes during right ventricular systole and diastole is depicted in Fig. 1 (top). High velocity regions develop in the IVC, SVC and RV outflow tract (RVOT) during the atrial filling phase (Fig. 1, top, left). Both inflow streams from the IVC and SVC lead to a rotational flow in the RA. In the later stages of ventricular systole, the caval venous flows meet each other, but do not exactly display a head-on collision in the RA (part of the IVC jet deviates towards the posterior atrial wall). In the diastolic phase after the closure of the PV, a high velocity region develops in the vicinity of the TV surrounded by rotational flow regions (Fig. 1, top, right). On the other hand, three- dimensional streamlines, color coded for vorticity magnitudes, show relatively low vorticity magnitudes along the IVC and SVC (Fig. 1, bottom), even though the spiraling streamlines correspond to the previously identified rotational regions (Fig. 1, top). The difference might come from the fact that vorticity describes the flow’s local rotationality whereas streamlines make larger scale flow circulations apparent. In the systolic phase, higher vorticity magnitudes are displayed in the proximity of the RVOT and RA, whereas during the diastolic phase high magnitudes develop in the vicinity of the TV.

### Vortex ring formation analysis

We now focused on vortex detection using the Q criterion, and analyze the temporal evolution of vortical structures in the RA.

**Figure 2:**
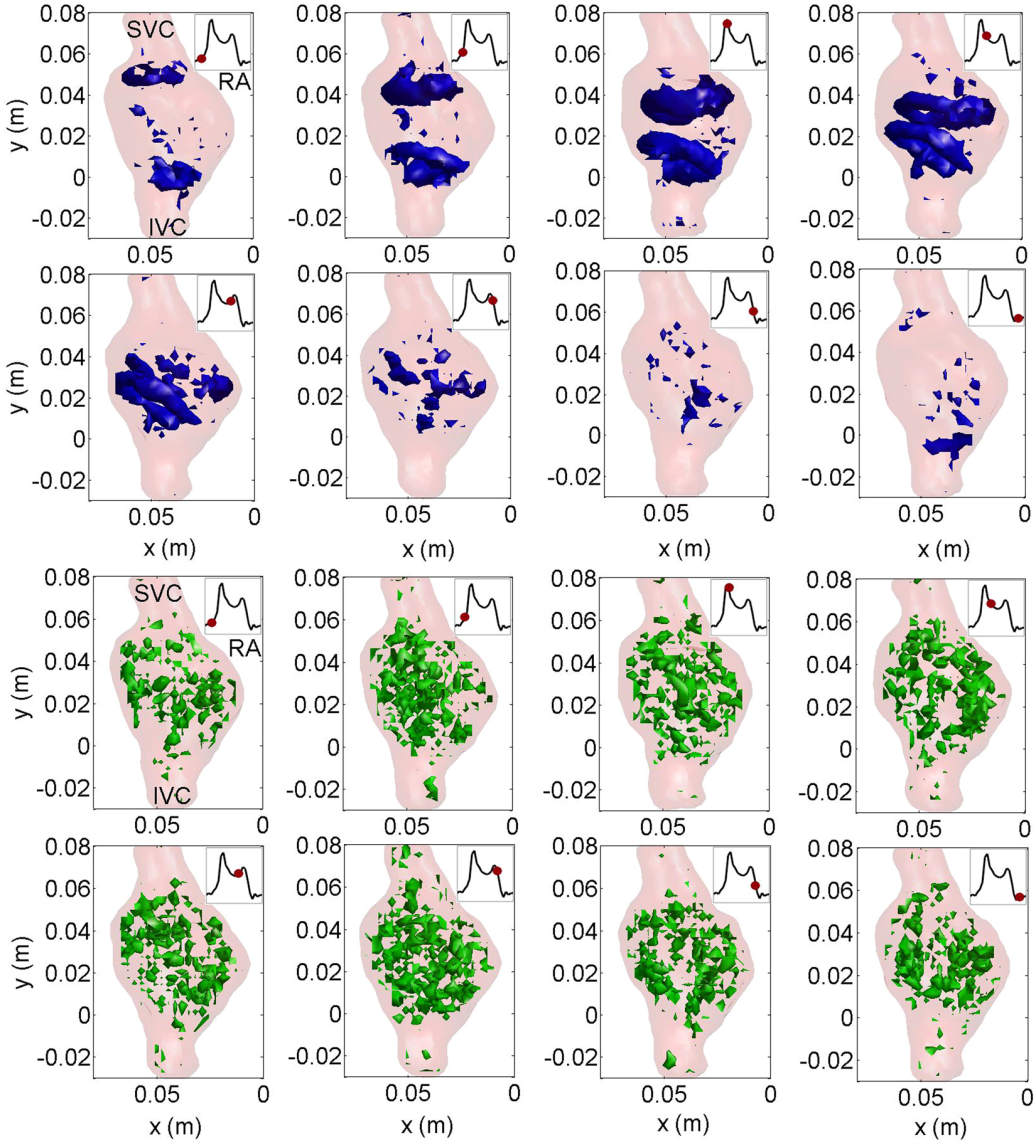
Time sequence of Q criterion-detected vortex structures in the phase averaged vorticity field (visualized as isosurfaces in blue color) and fluctuating vorticity field (visualized as isosurfaces in green color) over the entire cardiac cycle. Time instances are depicted as insets with a red circle (isovalue is 50 s^-1^).

In Fig. 2, both phase averaged and fluctuating vortical structures are shown for eight subsequent time instance along the duration of the pulse (phase averaged vortex structures are color-coded with blue color in the top panels, whereas the fluctuating structures are color-coded with green color in the bottom panel of Fig. 2). In the phase averaged field, two vortex rings are clearly visible in the vicinity of the transitional region from the IVC and SVC to the RA at the beginning of atrial filling. The vortex-rings grow during the atrial filling stage. Their size increases considerably prior to the collision of the two vortices and they are large enough to rinse the posterior atrial wall. In the ventricular filling stage, the vortex-rings break down. On the other hand, the fluctuating vortical structures are present during the entire cardiac cycle. While the phase averaged vortical structures are large with a size comparable to the diameter of the atrium, the fluctuating ones are small-scale structures.

**Figure 3:**
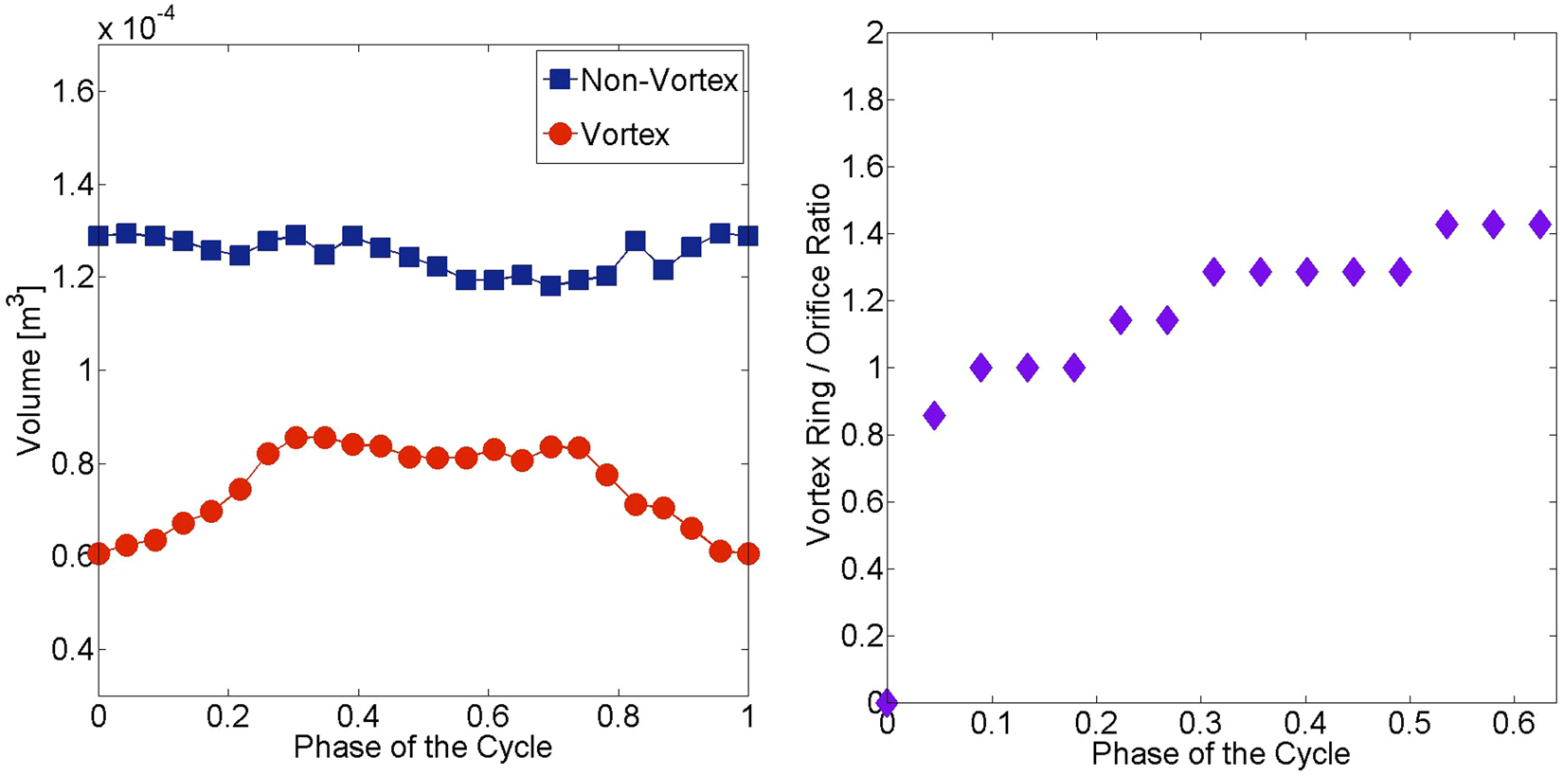
Time variation of vortex and non-vortex volume (left) and temporal evolution of the ratio between the vortex ring diameter and the diameter of both venae cavae (right).

Relevant vortex ring parameters are depicted in Fig. 3. The vortex ring grows over time and hence the volume of the vortical structures increases in the atrial filling phase (87 ml at peak systole). Disconnected parts of the vortical structures still exist after the vortex break down but they decay and their volume decreases until the end of the cardiac cycle (Fig. 3, left). As visible in Fig. 2, the coherent vortex rings break down in the early ventricular filling phase and we plot the vortex ring to orifice ratio until that moment in Fig. 3 (right). The vortex size is initially very similar to the venae cavae sizes and grows to a maximum value of 1.4 which agrees with *in vivo* study of Arvidsson investigating the healthy heart^5^.

### Wall shear stress (WSS) analysis

**Figure 4:**
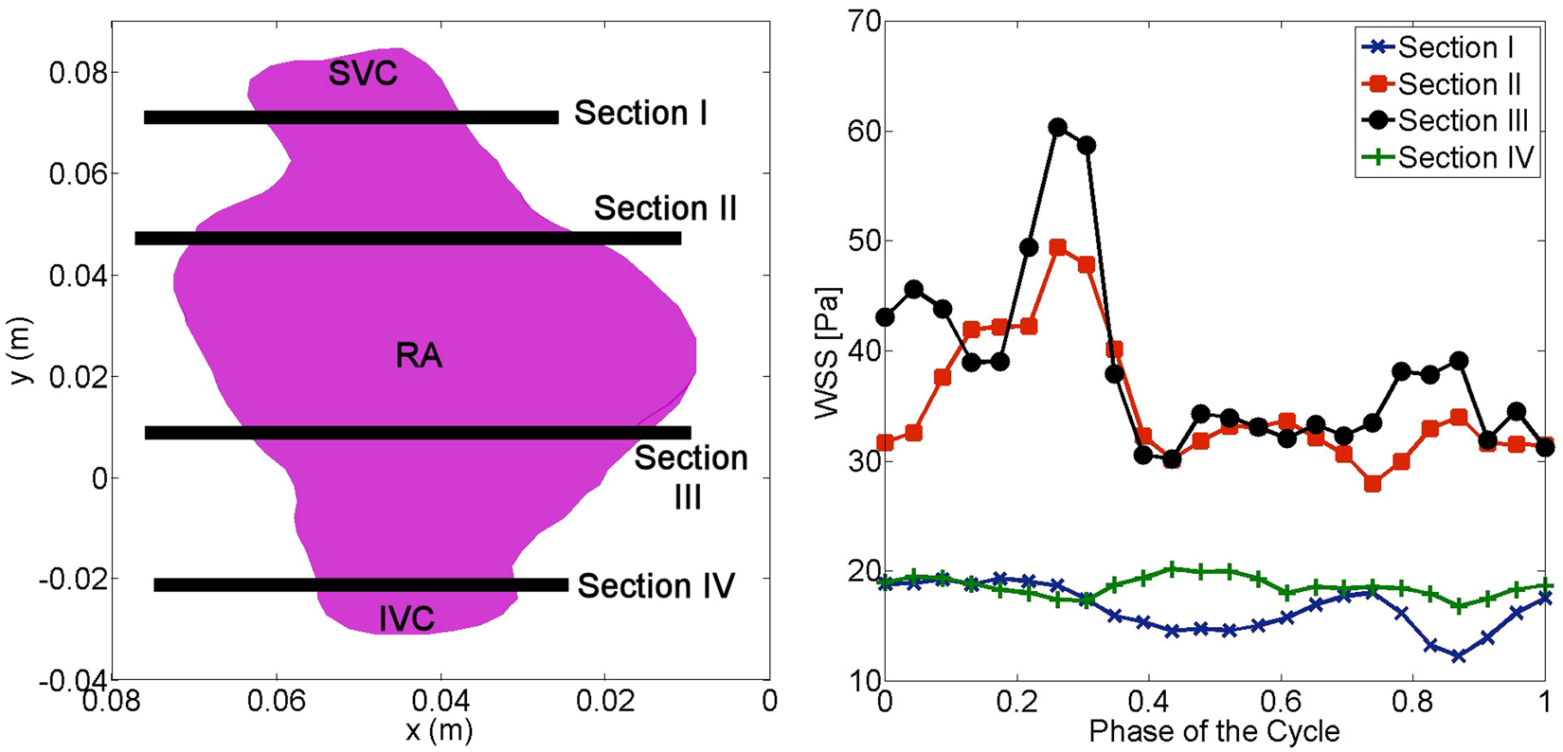
Cross sections investigated along the SVC, IVC and RA (left), time variation of spatially averaged WSS at four cross sections (right).

The temporal evolution of spatially averaged WSS at different cross sections (a) is depicted in Fig. 4(b). The time variation of WSS is minor at both venae cavae. However, the temporal trend of WSS in the RA (Section II and III) is different from the IVC and SVC, i.e. it varies significantly time. As seen, the WSS increases in the beginning of the atrial filling phase which corresponds to the time instant when the vortex rings are formed. In the late systole, the magnitude of WSS decreases and remains smaller during the diastolic phase. In the absence of the coherent vortex rings, e.g. diastolic phase in the RA and entire cardiac cycle in the IVC and SVC, the magnitude of WSS is comparatively smaller. It does seem that the presence of the vortex rings increases the WSS.

**Figure 5:**
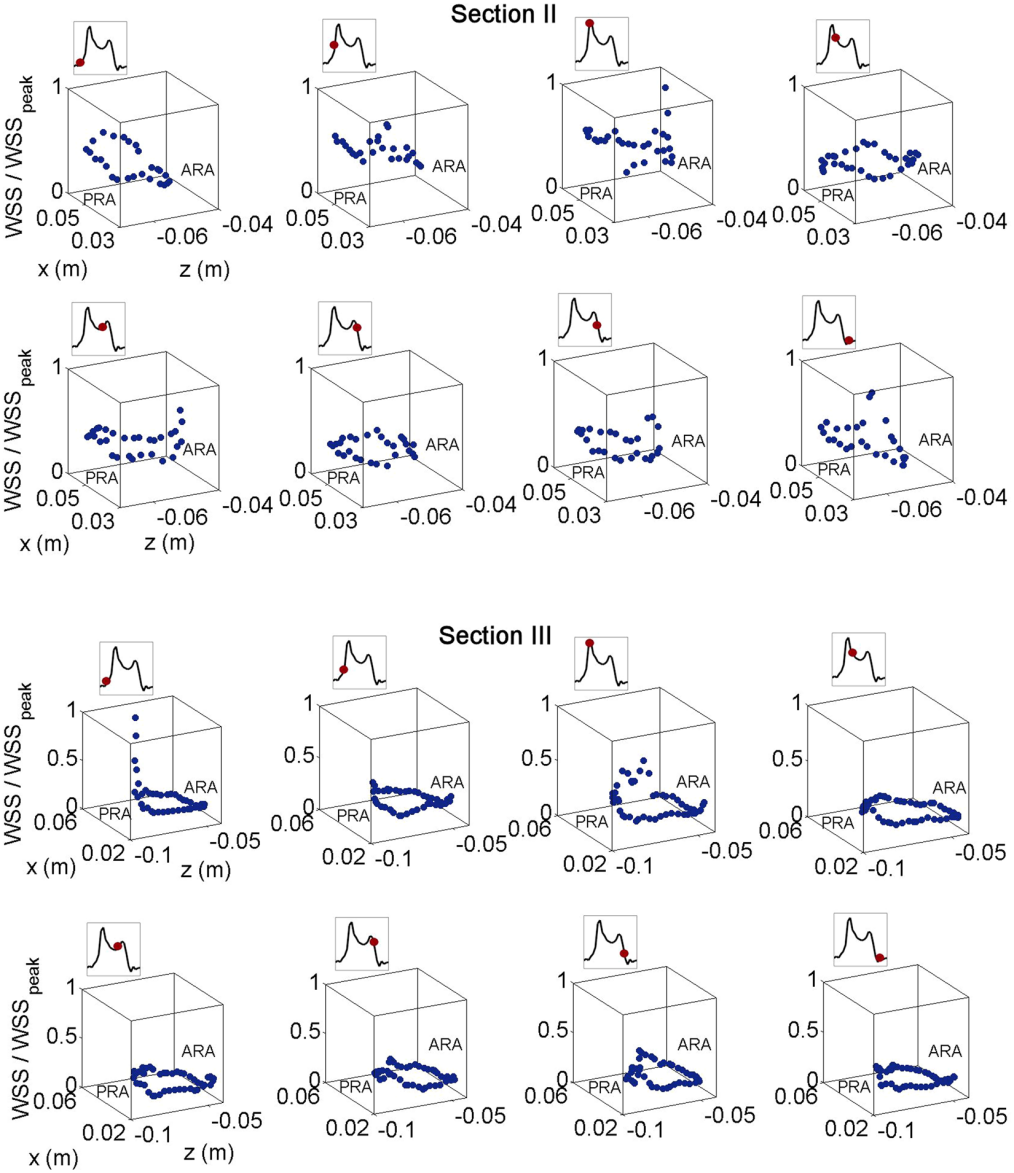
WSS normalized by peak WSS distribution along the RA circumference at Section II and III for eight different time instances (PRA represents posterior right atrium, ARA represents anterior right atrium).

We substantiate this relationship by focusing on the spatial distribution of WSS at the cross sections shown in Fig. 5. As seen at Section II, higher WSS regions are found at the posterior right atrium (PRA) in the atrial filling phase. During the ventricle filling stage, higher WSS regions are located near the anterior right atrium (ARA), i.e. in the proximity of the tricuspid valve. The spatial distribution of WSS at Section III is similar to the one at Section II. Higher WSS regions develop at the posterior right atrium.

### Statistical analysis

**Figure 6:**
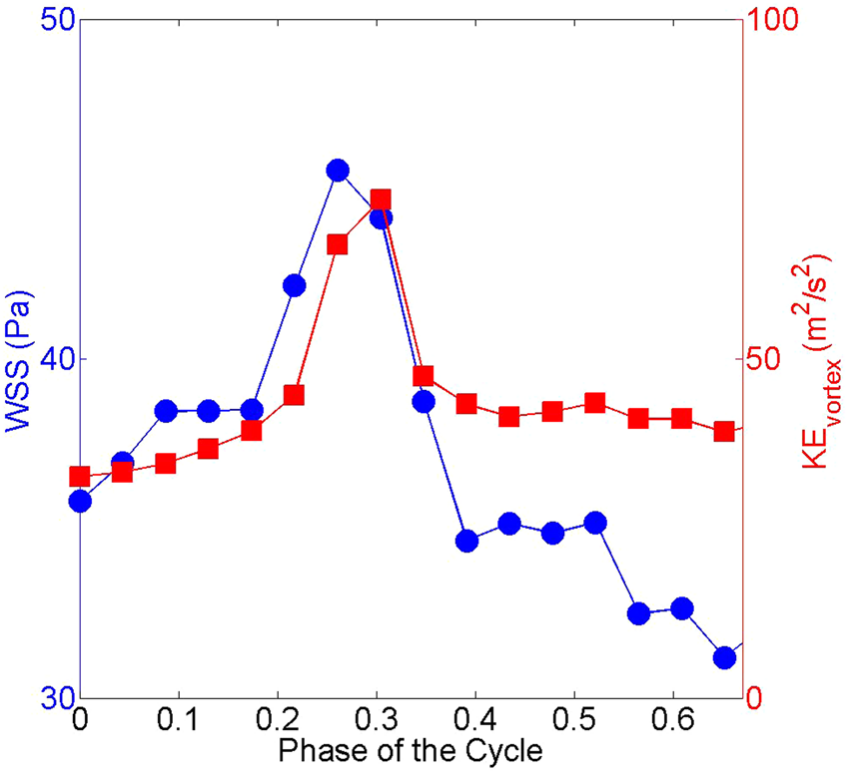
Time evolution of spatially averaged WSS (blue circles) and kinetic energy of the vortex ring (red squares).

In the previous sections, we qualitatively show that the vortex ring and WSS correlates well. We now focus on the statistical analysis between the vortex formation and WSS in Fig. 6. As seen (Fig. 6), time evolution of WSS and vortex kinetic energy shows same trends, i.e. an increase in the early atrial filling stage and a decrease in the late systolic phase. There is a correlation between WSS and the kinetic energy of vortex which is quantified based on a Pearson correlation coefficient of 0.7. This finding also shows that tracking the temporal behavior of the vortex structures may provide a complimentary information on the WSS as they have similar time trends.

## Discussion

We present an investigation which, for the first time, analyzes right atrial flow patterns in an *in vitro* silicone replica of the healthy human right heart by 3D PTV under physiological pulsating flow conditions. Time evolutions of vortex structures and associated shear stresses have been studied to characterize blood flow in the RA. The main findings of the present study are as follows.

1. Two vortex rings develop from the both venae cavae in the early atrial filling phase. The vortex rings grow during the ventricular systole and break down at the early right ventricular diastole.
2. The volume of the vortex structures at peak systole covers 40% of the entire RA volume. Furthermore, the vortex ring to orifice ration reaches to 1.4 prior to the break down which means that vortex rings grow and fill completely the atrial cross section.
3. It is shown that there is a correlation ($\rho =0.7$) between the vortex formation and the WSS. During the ventricular diastolic phase in the RA and entire cardiac cycle in the IVC and SVC, the magnitude of WSS is smaller when there is no coherent vortical structure formed. On the other hand, WSS becomes about 3 times higher due to the presence of the vortex rings during the ventricular systolic phase.
4. The qualitative analysis of the flow field revealed that the vortex rings provide rinsing effect along the posterior atrial wall which may avoid the risk for thrombus formation as the blood cells are washed away.

From a fluid mechanics point of view, recirculating flows and vortices are characteristic flow features in between the cardiac chambers, which play a crucial role in the momentum transfer and irreversible energy loss^14,15^. In healthy human hearts, the intraventricular flow pattern has a natural structure that is optimal for minimizing the energy dissipation^16^. An increase in energy dissipation due to the break of the natural structure may lead to an increase in the energy that is needed by the myocardial muscle to eject the blood into the circulation, which also increases myocardial oxygen consumption^16^. It is known that vortex rings optimize the blood flow within the heart^14,17^. We show in the phase averaged field that coherent vortical structures arise in the RA during the atrial filling stage which may transport the momentum efficiently reducing the energy loss.

RA thrombosis is a rare phenomenon, and has been reported under specific circumstances such as endocardial pacing^9^, and the condition is likely underdiagnosed^10^. Significant tricuspid regurgitation, low cardiac output, pulmonary hypertension and decreased pulmonary blood flow are predisposing parameters^18,19^. In some clinical cases, it is found that RA thrombi are in transit, having migrated from the venous system to the heart;^10,18^ which may predispose to pulmonary embolism. Right atrial enlargement in cardiovascular diseases affecting the right-sided cardiac chambers may also predispose to RA thrombus formation due to loss of atrial contractility, atrial arrhythmias such as atrial fibrillation and finally blood stasis. ARVC is a genetic myocardial disease that primarily affects the right ventricle, which is characterized by dilatation of the right ventricle wall motion abnormalities, as well as aneurysms. It is a common cause of sudden cardiac death in the young and athletes due to rapid ventricular arrhythmias. However, recently, it has become evident that the RA is commonly involved in the disease process leading to atrial arrhythmias^20^. In ARVC, RA dilation frequently occurs due to significant tricuspid regurgitation, RA volume and pressure overload, and atrial arrhythmias^21^. In some case studies, it is suggested that RA thrombosis needs to be recognized as a complication of ARVC^22,23^.

With regard to this, our results show that – even in the healthy human heart - low shear stress zones arise within the anterior RA in the absence of coherent vortical structures, which may be potential risk regions for development of thrombi, as low wall shear stress can provoke intimal wall thickening^24^. Our findings are in line with the case study showing a solid thrombus was attached to the free wall of the right atrium^22^. Recently, Parikh *et al*.^25^ qualitatively studied the right atrial flow patterns in the normal heart and in patients with patent foramen ovale (PFO). They found that vortical flow exist in the structurally normal heart, whereas the absence of the “standard” vortex formation during the atrial filling is more common in the patients with PFO. This *in vivo* study supports our findings on the vortex formation in the healthy right atrium.

Platelet adhesion and aggregation are responsible for triggering the thrombus formation mechanism and activated platelets might adhere to the thrombogenic surface at low WSS regions^26^. From a clinical point of view, low blood flow and stasis are risk factors for thrombosis. As shear rate increases, advective effects become dominant preventing thrombin production on the subendothelium^27^. Based on the findings from this study, we hypothesize that the influence of rinsing motion of the large vortex rings during atrial filling increases the WSS and thereby avoids RA thrombus formation. This assumption well agrees with clinical research on shear rate related thrombosis.

Limitations: We investigated flow patterns in a physiological right heart model, which is however obtained from a real patient geometry and driven with realistic forcing. Nevertheless, clinical studies on the differences in the formation of vortical structures in patients with right heart diseases, such as ARVC are still missing and this is subject of future work.

## Methods

We confirm that all methods were carried out in accordance with relevant guidelines and regulations. This study was conducted in full agreement with the principles of the “Declaration of Helsinki” and current Swiss law. It has been approved by the Ethics Committee of the Canton of Zurich (approval number KEK-ZH-Nr. 2014-0443). All probands signed an informed consent form for prospective inclusion.

### Anatomic reconstruction of the right heart

**Figure 7:**
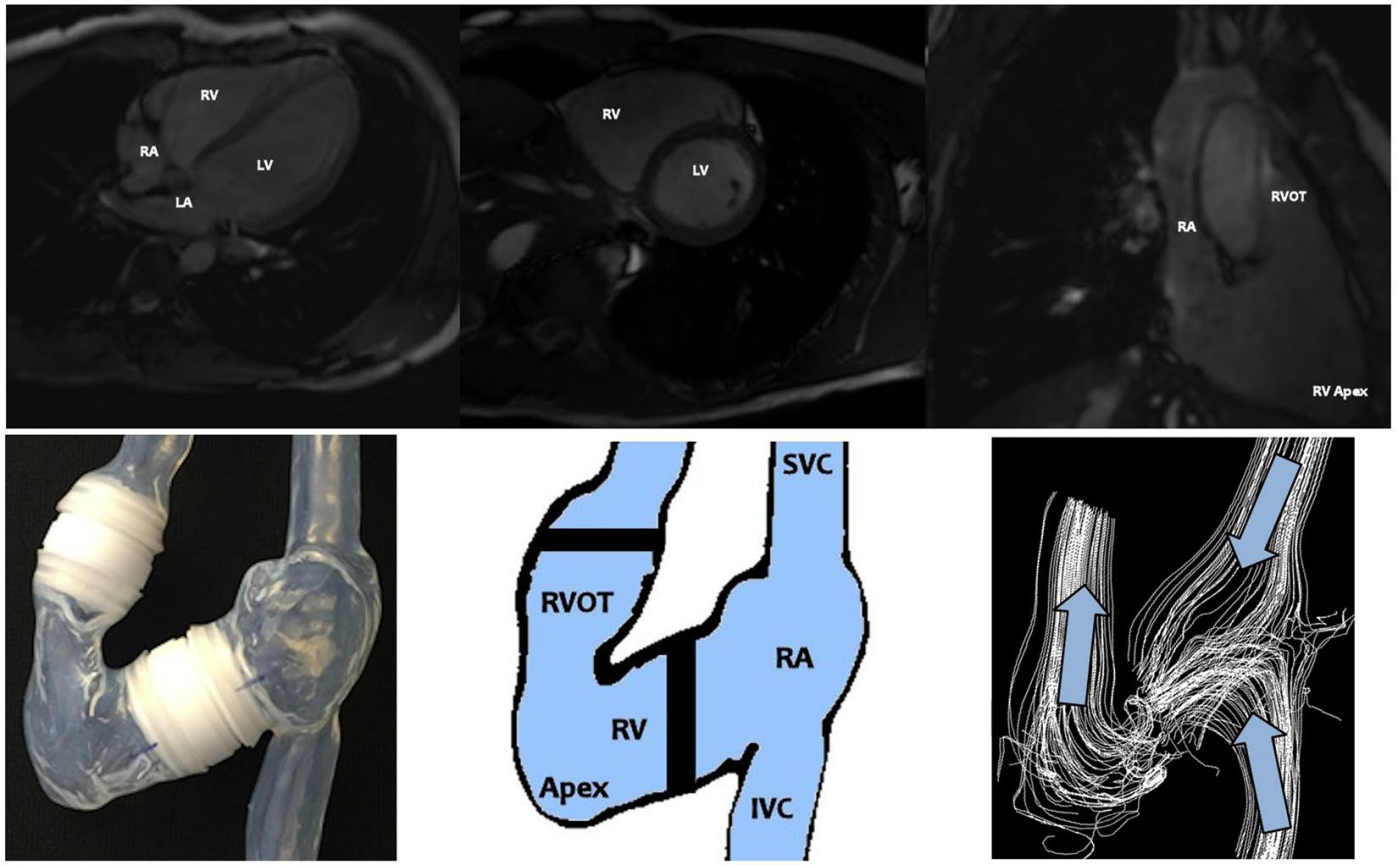
4 chamber view (top, left), RV short axis view (top, middle), RV long axis view (top, right) by *in vivo* magnetic resonance imaging (MRI) of a healthy male heart; image of the compliant *in vitro* silicone phantom (bottom left), region of interest and parts of the right heart (bottom, middle), qualitative visualization of the flow streamlines (white color-coded) and two inflow and one outflow regions (blue colored arrows) within the right heart. (IVC: inferior vena cava, RA: right atrium, RV: right ventricle, RVOT: right ventricular outflow tract, SVC: superior vena cava).

ECG-gated cardiac-magnetic resonance imaging (MRI) with 1.5 Tesla (Philips, The Netherlands) was performed in a healthy adult male aged 25 years in order to obtain *in vivo* time-resolved anatomic maps of the right atrium (RA), superior vena cava (SVC), inferior vena cava (IVC), and RV including the RV outflow tract (RVOT) (Fig. 6, top). The blood enters the right heart via the SVC and IVC (Fig. 7, bottom). The two inflows mix in the right atrium, which acts as a reservoir for systemic venous return when the tricuspid valve (TV) is closed, whereas it acts as a passive conduit when the TV is open, and acts as an active conduit during atrial contraction to assist in RV filling^28^.

### 3D-PTV measurements

**Figure 8:**
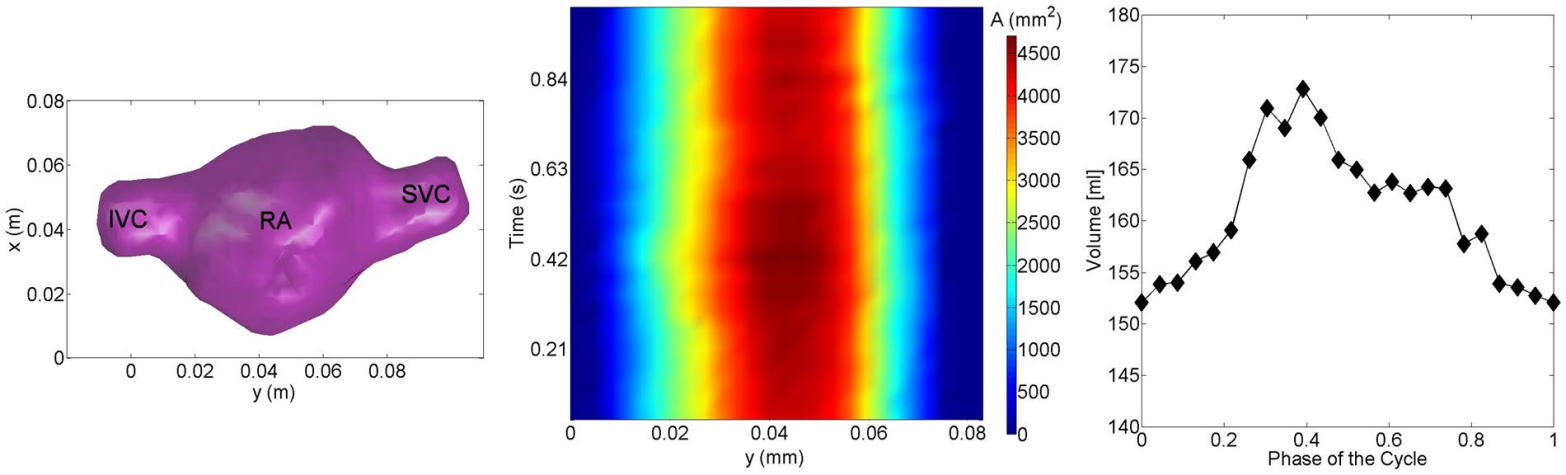
The right heart geometry including IVC, SVC and RA extracted from 3D-PTV measurements (left), time variation of cross-sectional areas for different cross-sections (middle), and right atrial volumetric variation (right).

3D Particle Tracking Velocimetry (3D-PTV), an optical imaging tool based on tracing neutrally buoyant particles in the flow, has been applied to an anatomically accurate silicone replica (Elastrat GmbH, Switzerland) of the human RV and RA derived from an *in vivo* MRI heart scan of a healthy male proband. Other investigators and in our own work, we have previously used 3D-PTV to assess turbulence related quantities such as turbulent kinetic energies (TKE), and irreversible energy loss in the ascending aorta *in vitro* ^29–31^. In this study, the investigation domain for 3D-PTV measurements comprises the RV, RA, IVC and SVC (Fig. 8, bottom). Two prosthetic mechanical valves (Regent 25 *mm*, St. Jude Medical, St. Paul, USA) were implemented at the pulmonary valve and tricuspid valve positions. The pulsatile flow along the region of interest (ROI) is provided by a pump system, which is composed of an external pressure chamber, i.e. an 80 ml-ventricular assist device (VAD, MEDOS, Germany), and a waveform generator, i.e. an electro-pneumatic pump (Berlin Heart, Germany). The heart model allows active expansion and contraction of the right ventricle using an external pressure chamber, which results in physiological flow conditions. The atrium expands in the atrial filling phase by means of the inflows from both venae cavae, whereas it contracts in the ventricular filling phase (Fig. 8). The chamber volumes of this model are similar to those in clinical studies^8^.

3D-PTV is an optical imaging tool and its main principle is to track tracer particles illuminated with a light source with a high-speed camera. In our experimental setting, a high-speed camera (Photron SA5, Japan), which allows recording 1.56 seconds at full resolution of 1024 × 1024 pixels and 7000 frames per second, with a Nikkon AF Micro Nikkor 60 *mm* f/2.8 D lens (Japan) was used to capture images during flow. The high-speed camera was synchronized with the heart pump system to trigger the recordings at the beginning of every cardiac pulse. As a light source, a diode-pumped Nd-YLF laser (Quantronix, Darwin Duo 527 *nm*, USA) was used to illuminate the investigation domain. As tracer particles, fluorescent rhodamine particles (Cospheric, USA) with a diameter of 200 *μm* were used^32^. It is important to avoid optical distortions due to index of refraction mismatch between the silicone material and the working fluid for the 3D-PTV measurements. To overcome optical distortions, a mixture of glycerine, water and sodium chloride was used as working fluid, which matches the refractive index of silicone. The kinematic viscosity is $4.85\times {10}^{-6}{m}^{2}/s$ which is similar to the one of the blood at high shear rates. The position accuracy of the particles was 0.17, 0.17 and 0.35 *mm* in x, y and z directions, respectively. The velocity uncertainty of the raw PTV data, as obtained from the calibration, was 0.038 *m*/*s*. As for the velocity assigned to a voxel and after smoothing, the final accuracy was estimated to be 4 × 10^−4^ *m*/*s* ^32^. The investigation domain covers the RA, SVC and IVC. A total of 36 cycles were recorded to obtain a phase averaged flow field. The measurements comprise around 105 images corresponding to a total number of 108 particle positions, which allows obtaining converged statistics for phase averaged flow fields and velocity fluctuations. The RV stroke volume was 77 *ml* and the heart rate 50 beats per minute, resulting in a physiologic cardiac output of 3.9 *l*/*min* at resting conditions.

### Vorticity identification

Vorticity is the curl of the velocity field. The phase averaged vorticity is defined as ${\rm{\Omega }}=\nabla \times {U}_{i}$ obtained from the phase averaged velocity, *U*_*i*_, whereas the fluctuating vorticity field is defined as $\omega =\nabla \times {u}_{i}$ obtained from the fluctuating velocity, *u*_*i*_. The Q criterion, which identifies vortices if the norm of the local rate of rotation tensor ($\Vert R\Vert$) is dominant over the norm of the local rate of strain tensor ($\Vert S\Vert$)^33^, is used for the identification of coherent rotational structures in the phase averaged and fluctuating velocity fields. The Q criterion is defined as

1 $$Q=1/2({\Vert R\Vert }^{2}-{\Vert S\Vert }^{2}) > 0$$

where $R=\mathrm{1/2(}\nabla {U}_{i}-{[(\nabla {U}_{i})]}^{T})$, $S=\mathrm{1/2(}\nabla {U}_{i}+{[(\nabla {U}_{i})]}^{T})$ for the phase averaged velocity. The same definition using *u*_*i*_ instead of *U*_*i*_ is applied for the fluctuating fields.

### Wall shear stress analysis

The investigation of wall shear stress is of high interest as it has been suggested to represent an indicator for thrombosis formation^24^. The total wall shear stress (WSS), which includes both viscous and turbulent WSS, is defined as

2 $$\tau ={\tau }_{vis}+{\tau }_{turb}$$

The viscous WSS at a certain time instant is a tensor calculated as

3 $${\tau }_{vis}=\mu (\delta {U}_{i}/\delta {x}_{j})$$

where *μ* is the dynamic viscosity of the fluid.

The turbulent WSS tensor is defined as

4 $${\tau }_{turb}=-\rho ({u}_{i}^{\prime} {u}_{j}^{\prime} )$$

where *ρ* is the density of the fluid and $({u}_{i}\text{'}{u}_{j}\text{'})$ are the Reynolds stresses. We use the L2 norm of the WSS tensors to quantify WSS magnitudes.

As shown in Fig. 7, the measurements were performed in a transparent, compliant silicon model in our *in vitro* study where the location of the walls is not known a priori. We extracted the geometry of the atrium from our 3D-PTV measurements. Firstly, we transformed the volumetric velocity data into a binary data by assigning to each voxel “1” if there is velocity information (meaning that the voxel is part of the flow domain in the atrium) or “0” if the voxel if there is no velocity information (meaning that the voxel is in the atrium walls or outside the atrium). Secondly, we set an iso-value of 0.99 (Fig. 8, left) to the binary field to reconstruct the walls of the atrium geometry. The reconstruction is not sensitive to the particular choice of the isovalue between zero and one because the transition from zero to one is very sharp. The error in the estimation of the wall location is of about 250 *μm*.

An accurate assessment of viscous wall shear stresses is challenging as it needs a very fine spatial resolution comparable to the length scale of the viscous sublayer. The size of the viscous layer is estimated around 60 μm, which is more than an order of magnitude smaller than the voxel size (1 *mm*) However, we can make use of the fact that the total wall shear stresses decrease slowly over the transition between viscous sublayer and turbulent layer where the viscous shear is gradually replaced by the turbulent WSS component^34^. Even though we measure shear stresses at a finite distance from the wall the sum of viscous and turbulent shear stresses are therefore representative to the effective shear stresses at the wall.

**Figure 9:**
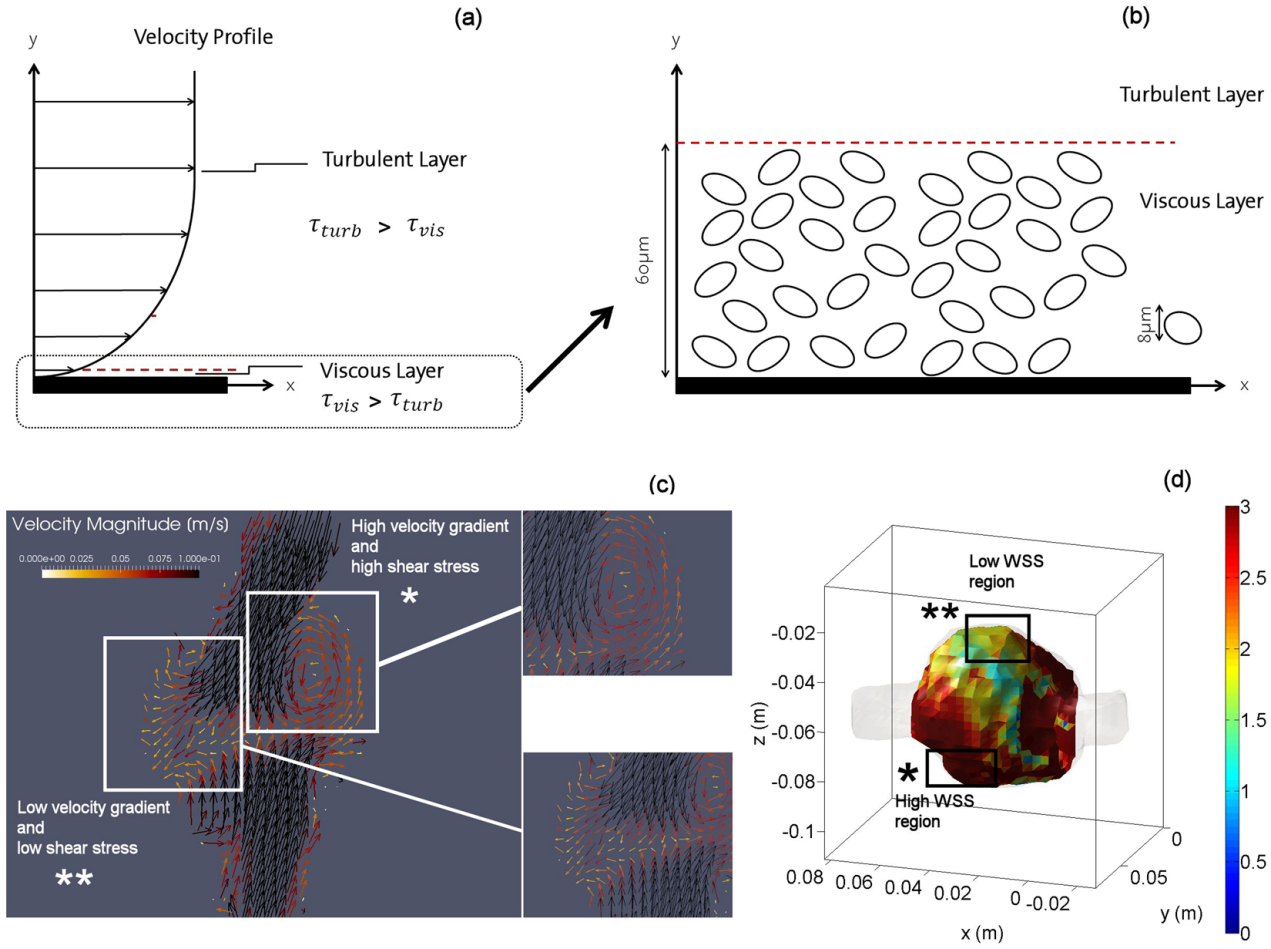
Schematic of flow velocity profile and shear stresses (**a**), transport of platelets and the red blood cells in the viscous layer (**b**), phase averaged velocity vector map highlighting the vortical and non-vortical regions (**c**) and associated WSS (**d**). The blue color in (**d**) corresponds to low WSS regions and the red color corresponds to high WSS regions.

The wall shear stresses are calculated using a velocity interpolation to positions with fixed normal distance to the detected boundary. We achieve a resolution of O(1 mm) in our measurements. Depending on the spatial resolution, the WSS may vary, i.e. fluctuating WSS become dominant outside the viscous layer, whereas they are zero at the wall (Fig. 9a). The velocity pattern and WSS relation in the vortex and non-vortex regions are qualitatively shown in Fig. 9(c,d). Fluid velocity and its spatial gradients are typically higher in proximity of the vortex resulting in a high WSS region whereas the velocity tends to be more uniform in the regions where no vortex is present.
